# Endovascular Treatment of Isolated Iliac Artery Aneurysms with Anaconda Stent Graft Limb

**DOI:** 10.1155/2013/527492

**Published:** 2013-06-19

**Authors:** Christos Karathanos, Elias Kaperonis, Dimitrios Xanthopoulos, Theophanis Konstantopoulos, Maria Exarchou, Caterini Loupou, Vassilios Papavassiliou

**Affiliations:** Department of Vascular Surgery, Sismanoglio, General Hospital of Athens, Sismanogliou 1, Marousi, 15126 Athens, Greece

## Abstract

Isolated aneurysms of the iliac arteries are relatively rare conditions that traditionally have been treated by surgical reconstruction. We report our experience with endovascular treatment of iliac artery aneurysms (IAAs) with Anaconda stent graft limb. Two male patients were found to have 4.5 and 3.6 cm isolated common IAAs, respectively. The endograft was successfully advanced and deployed precisely to the intended position in both cases. In one case the internal iliac artery was embolized. No type I or II endoleak was observed immediately after the procedure. In one patient postimplantation fever (>38°C) and gluteal claudication occurred. After 2 years followup both iliac endovascular stent grafts are patent and without endoleak. Endovascular treatment with Anaconda limb stent graft seems to be a safe and feasible alternative to open surgery.

## 1. Introduction

Isolated aneurysms of the iliac arteries are relatively rare representing 2% to 7% of all intra-abdominal aneurysms [1, 2]. Iliac artery aneurysms (IAAs) are most frequently localized in the common or internal iliac artery (IIA) in combination with an abdominal aortic aneurysm. Aged men are most commonly affected and the etiology is usually atherosclerotic. 

Most of the patients with IAAs are asymptomatic and diagnosed incidentally. Elective repair has been recommended for IAAs with diameter greater than 3 cm to reduce the risk of rupture. Traditionally they have been treated by surgical reconstruction [3, 4]. The development of new endovascular devices offers alternative therapies [5]. 

We report our experience in endovascular treatment of isolated iliac artery aneurysms with the Anaconda limb stent graft.

## 2. Case Reports

### 2.1. Case 1

**Figure 1:**
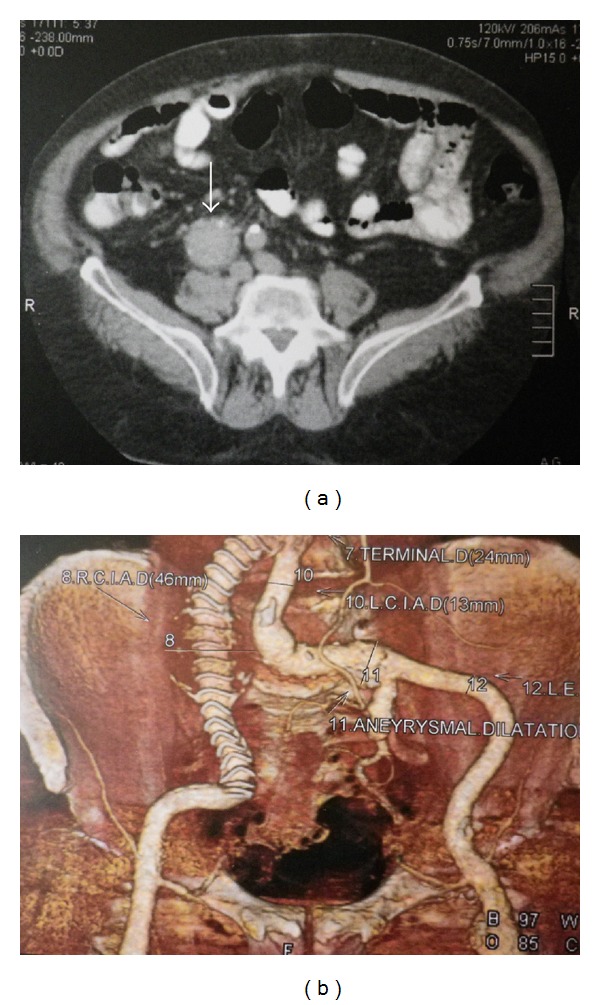
(a) Preoperative contrast-enhanced abdominal CT showing severe aneurysm of the RCIA with diameter 4.5 × 4.7 cm. (b) Contrast-enhanced CT scan reconstruction at 18 months showing patency of the endograft, complete aneurysm exclusion without any endoleak.

A 74-year-old obese man with chronic obstructive pulmonary disease, hyperlipidemia, and extensive smoking history was referred to our department because of an aneurysmal dilatation of the right common iliac artery (RCIA) detected on an abdominal ultrasound. A contrast-enhanced abdominal computed tomography (CT) demonstrated an aneurysm of the RCIA with diameters of 4.5 × 4.7 cm (Figure 1(a)). The length of the aneurysm neck proximally was 18 mm and the ipsilateral IIA was thrombosed. The external iliac artery (EIA) was 13 mm in diameter with mild calcifications and tortuosity.

In an operating room equipped with C-arm fluoroscopy under epidural anesthesia we surgically exposed the right common femoral artery (CFA) through an incision 1-2 cm above and parallel to the inguinal crease. A 5000-unit amount of heparin was given intravenously before endovascular manipulations. The ipsilateral CFA was punctured, an 8-F sheath was introduced, and an angiography was performed. Under fluoroscopy soft angled tip 0.035 inch wire and catheter were passed into the abdominal aorta. The wire was exchanged for a stiff guide wire with a soft tip and the delivery system of the Anaconda limb was advanced in the iliac system. A straight self-expandable Anaconda limb stent graft (Vascutek Ltd., Inchinnan, Renfrewshire, Scotland), 18 mm diameter by 140 mm length, was deployed to the RCIA and EIA. Completion angiography through the 8F sheath repositioned in the common iliac artery showed no type I or II endoleak. Once all materials were removed, closure of the artery was achieved with 3 stitches and wound was closed in standard fashion. Radiation time was 4 min and the contrast volume used was 50 mL. The patient tolerated the procedure well and was discharged under long-life therapy with aspirin and statins on the third postoperative day. 

A postoperative contrast-enhanced CT scan at 1, 6, and 12 months was performed showing reduction in the size of the aneurysm and no endoleak or migration (Figure 1(b)).

### 2.2. Case 2

**Figure 2:**
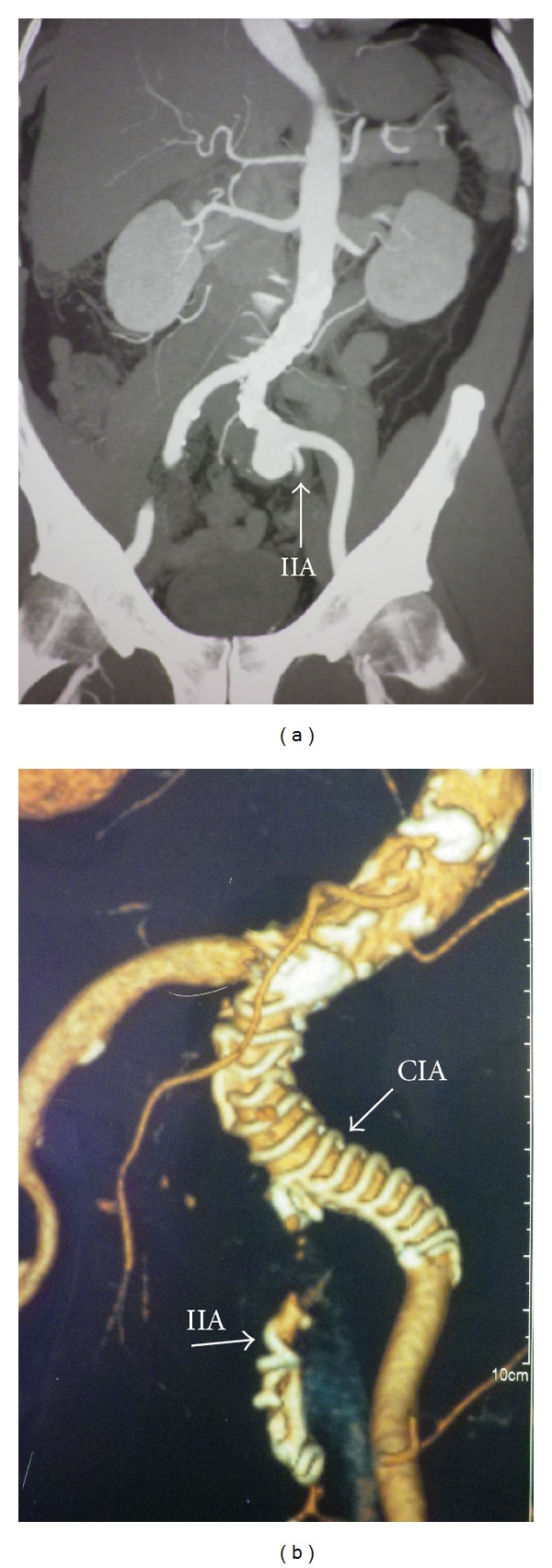
(a) A 60-year-old male patient with LCIA aneurysm with diameter 3.5 × 3.6 cm, length 3.8 cm, and severe angulation of the iliac axis extended to the orifice of the IIA. (b) Two years postoperatively, the aneurysm sac is thrombosed. Notice the coils into the IIA.

A 60-year-old man presented with a palpable pulsatile mass in the left iliac fossa. Contrast-enhanced abdominal CT demonstrated an aneurysm of the left common iliac artery (LCIA) with diameter 3.5 × 3.6 cm, length 3.8 cm, and severe angulation (>60°) of the iliac axis. The patient had a history of hypertension, hyperlipidemia managed by medical treatment, a positive family history for coronary artery disease, and he smoked 30 cigarettes a day. The aneurysm extended to the orifice of the IIA. Coil embolization of the left IIA was decided to prevent backfilling of the aneurysm (Figure 2(a)) (the contralateral IIA was patent). The proximal neck of the aneurysm was 20 mm in diameter and the diameter of the CFA was 11 mm. A similar surgical procedure to the previous case was conducted under epidural anesthesia. Embolization of the IIA with 4 coils (Cook, MReye) was performed first and then a straight Anaconda limb stent graft 15 × 80 mm extending from the CIA to the EIA was successfully deployed. Final angiography revealed an optimal result without any endoleaks. The procedure was uneventful, and radiation time and contrast volume used were 6 min and 70 mL, respectively.

On day 2 after surgery, the patient developed a fever >38°C with mild leucocytosis and elevation of the C-reactive protein level consistent with postimplantation syndrome. This settled spontaneously after 2 days. The patient was discharged on the sixth postoperative day under antiplatelet therapy with aspirin. During this postoperative period the patient reported new onset of buttock claudication, but these symptoms were completely resolved in the first postoperative visit at 1 month. The patient remained asymptomatic and without any graft-related complications at 2 years of followup (Figure 2(b)).

## 3. Discussion

Iliac artery aneurysms most commonly coexist with aneurysms of the abdomen. Isolated IAAs are relatively uncommon with an incidence of 0.03% in autopsy series [4, 6]. Most IAAs are atherosclerotic while other causes include trauma, infection, mycosis, dissection, anastomotic after previous arterial reconstruction, excessive athletic effort (bicycle racing), and collagen disorders such as Marfan and Ehlers-Danlos syndromes [3]. Usually IAAs are asymptomatic and diagnosed incidentally, but they can become symptomatic as a result of compression on or erosion of adjacent structures, rupture, distal embolization, or thrombosis [4, 5, 7].

Despite their rarity, IAAs have a high risk of rupture with an associated high mortality rates of up to 80% [8]. Elective open surgical repair with aneurysm resection and graft interposition is indicated in the majority of the patients although this is a major intervention with considerable morbidity and mortality rates. Reported complications include ischemia of the lower extremities due to distal embolization or stenosis, colorectal and pelvic ischemia from disruption of the flow to the IIA, ureteral and iliac vein injury, hemorrhage, anastomotic aneurysm, arterioenteric fistula, and graft infection. Overall mortality rates ranges from 11% to 33% [3, 4, 9, 10]. Minimally invasive endovascular repair with a stent graft for aneurysm exclusion is an alternative treatment [9, 11, 12].

Endovascular repair of iliac aneurysms was first described in 1995 [13, 14]. Endovascular treatment options for isolated IAAs include aortoiliac endografts, covered stents, limbs of endovascular grafts for AAA repair, or iliac branched stent grafts. The diameter of the proximal sealing zone is often significantly larger than the diameter of the distal sealing zone and so a funnel-shaped stent graft is usually required. Iliac arteries are often tortuous and a flexible stent graft that can adapt to iliac anatomy is preferable [15]. The development of branched stent grafts for deployment into the IIA is another promising solution although anatomical conditions for their use are restricted. In some cases IAAs can be treated with aorto-bi-iliac or aorto-uni-iliac stent grafting to secure the stability of the graft and sealing of the aneurysm. Aorto-uni-iliac stent grafting requires an extra-anatomic bypass that increases the complications rate due to the presence of a foreign body.

In a systematic Medline search we identified all reported cases with endovascular treatment of isolated IAAs with commercially available stent graft limbs for AAA repair published between 1995 and 2012. Studies that failed to report the stent graft that was used, the surgical interventions, and outcomes were excluded. Only cases treated by endograft placement to unilateral iliac artery with or without IIA embolization were included. Technical success defined as successful completion of the endovascular procedure and no conversion to open was 100% [11, 16–22]. The 30-day mortality rate was <2% and overall primary patency rate exceeds 95%, with graft thrombosis being the most frequent cause. The most common complication was buttock claudication (16%) followed by type II endoleak (9.3%) and lower extremity ischemia (4.3%). Freedom from aneurysm related complications ranges from 85% to 100% and in most cases is related to endoleak from not embolizing the IIA. This stresses the importance of IIA embolization to prevent endoleak although there is a risk of ischemia from IIA flow disruption. 

Internal iliac artery embolization is not always a benign procedure. Bowel ischemia or buttock claudication to necrosis has been reported in several studies with an incidence of 10% to 30% [4, 15, 23, 24]. The risk of colonic mucosal ischemia becomes significant after occlusion of both IIA and exclusion of the inferior mesenteric artery (IMA) after endovascular repair of abdominal artery aneurysms (AAA). Angiography of superior mesenteric artery (SMA) should be performed first to evaluate collateral pathways to distal branches of the IMA. Buttock claudication is usually transient resolving within weeks explained by the fact that the lumbar, IMA, and deep femoral arteries may provide sufficient buttock collateral circulation after IIA embolization. The circulation provided by these collaterals appears not to be sufficient after AAA repair [25]. Embolization of the distal internal iliac branches has been suggested to increase the risk of pelvic ischemia, however, Boules et al. did not observe any difference in the incidence of buttock claudication in patients undergoing branch vessel versus main trunk IIA coiling [23, 25, 26]. Iliac branched stent grafts for preserving flow to IIA and reducing the incidence of pelvic ischemia is an alternative option [15].

 Iliac limb thrombosis may be caused by extrinsic compression from the contralateral iliac artery or docking zone and by incomplete expansion of the distal part of the iliac limb, in cases without severe angulation. In contrast graft thrombosis in patients with severe iliac angulation is usually due to recoiling of a calcified plaque at the distal portion of the iliac artery. Other complications of the endovascular treatment include lower limb ischemia due to technical failure or distal arterial embolization, access site complications including infection, hematoma, and pseudoaneurysms [24, 27]. 

 Anaconda limbs exist in straight or reversed flare configurations. When the diameter of the proximal sealing zone is significantly larger than the diameter of the distal sealing zone a reversed flare stent graft is therefore required. The reversed flare iliac leg is available in proximal diameters of 15, 17, 19, 21, and 23 mm, distal diameters of 12 and 17 mm, and lengths of 80 to 130 mm. The straight iliac leg is provided in diameters of 10 to 18 mm and lengths of 60 to 140 mm. This large size range of devices offers better options for varying patient anatomies.

In order to reduce the risk of graft-related complications the ideal endograft should be flexible, to adapts well to tortuous iliac anatomy and to warrant good proximal sealing. The multiple independent nitinol ring, externally supported by thin polyester, of the Anaconda iliac limb stent graft design provides maximum flexibility to cater for varying patient iliac anatomy and minimises the potential of kinking [28]. The proximal 1-2 rings of the stent graft can be placed just above the orifice of the CIA into the aorta, resulting in creation of a “double barrel” seal, which protects the device from migration. Furthermore, the distal part of the stent graft allows the in vivo length adjustment during deployment [19]. One possible disadvantage of the Anaconda iliac limb is that although the absence of longitudinal stents provides flexibility, this makes extrinsic compressions of the graft more likely and the risk of graft thrombosis greater. Despite this, no graft thrombosis was associated with Anaconda limb in all series. 

Power et al. reported 11 patients with 12 IAAs that were treated with Anaconda limbs [19]. There were no graft related complications although in our study one patient developed postimplantation syndrome. Saratzis et al. reported an incidence of post-implantation syndrome of 19% in 51 patients that were treated with Anaconda aortic stent graft for abdominal aortic aneurysm [29]. This further underscores the need to be highly suspicious of this syndrome that may prevent patient from discharge as it is usually self-limiting and resolves within one week without special treatment. In our series over a followup of 2 years the patients remained asymptomatic with no aneurysm-related complications. 

## 4. Conclusions

Open surgery repair of isolated IAAs is a highly invasive method with significant morbidity and mortality rates. Endovascular treatment with Anaconda limb stent graft seems to be safe and feasible, providing good mild term results. This endograft has good performance especially in iliac arteries with severe angulation; however, larger studies with longer followup are needed to establish the durability of such repair.
